# Sphingosine-1-Phosphate Receptor 1 Is Involved in Non-Obese Diabetic Mouse Thymocyte Migration Disorders

**DOI:** 10.3390/ijms19051446

**Published:** 2018-05-12

**Authors:** Julia P. Lemos, Salete Smaniotto, Carolina V. Messias, Otacilio C. Moreira, Vinicius Cotta-de-Almeida, Mireille Dardenne, Wilson Savino, Daniella A. Mendes-da-Cruz

**Affiliations:** 1 Laboratory on Thymus Research, Oswaldo Cruz Institute, Oswaldo Cruz Foundation, Rio de Janeiro 21040-900, Brazil; j.lemos@ioc.fiocruz.br (J.P.L.); cmessias@ioc.fiocruz.br (C.V.M.); vca@ioc.fiocruz.br (V.C.-d.-A.); savino@fiocruz.br (W.S.); 2 National Institute of Science and Technology on Neuroimmunomodulation, Oswaldo Cruz Institute, Oswaldo Cruz Foundation, Rio de Janeiro 21040-900, Brazil; smaniotto@icbs.ufal.br; 3 Laboratory of Cell Biology, Institute of Biological and Health Sciences, Federal University of Alagoas, Maceió, Alagoas 57000-001, Brazil; 4 Laboratory of Molecular Biology and Endemic Diseases, Oswaldo Cruz Institute, Oswaldo Cruz Foundation, Rio de Janeiro 21040-900, Brazil; otacilio@ioc.fiocruz.br; 5 French National Center for Scientific Research (CNRS), Mixed Research Unit (UMR) 8147, Paris Descartes University, 75006 Paris, France; mireille.dardenne@free.fr

**Keywords:** non-obese diabetic mice, thymus, sphingosine-1-phosphate, sphingosine-1-phosphate receptor 1, VLA-5 (very late antigen-5), cell migration

## Abstract

NOD (non-obese diabetic) mice spontaneously develop type 1 diabetes following T cell-dependent destruction of pancreatic β cells. Several alterations are observed in the NOD thymus, including the presence of giant perivascular spaces (PVS) filled with single-positive (SP) CD4^+^ and CD8^+^ T cells that accumulate in the organ. These cells have a decreased expression of membrane CD49e (the α5 integrin chain of the fibronectin receptor VLA-5 (very late antigen-5). Herein, we observed lower sphingosine-1-phosphate receptor 1 (S1P1) expression in NOD mouse thymocytes when compared with controls, mainly in the mature SP CD4^+^CD62L^hi^ and CD8^+^CD62L^hi^ subpopulations bearing the CD49e^−^ phenotype. In contrast, differences in S1P1 expression were not observed in mature CD49e^+^ thymocytes. Functionally, NOD CD49e^−^ thymocytes had reduced S1P-driven migratory response, whereas CD49e^+^ cells were more responsive to S1P. We further noticed a decreased expression of the sphingosine-1-phosphate lyase (SGPL1) in NOD SP thymocytes, which can lead to a higher sphingosine-1-phosphate (S1P) expression around PVS and S1P1 internalization. In summary, our results indicate that the modulation of S1P1 expression and S1P/S1P1 interactions in NOD mouse thymocytes are part of the T-cell migratory disorder observed during the pathogenesis of type 1 diabetes.

## 1. Introduction

NOD (non-obese diabetic) mice spontaneously develop type I diabetes (T1D) as a result of the autoreactive destruction of the insulin-producing β cells in the pancreatic islets. The NOD mouse strain provided a wealth of knowledge of the processes involved in autoimmune diabetes etiology and is one of the most used mouse models due to the similarity with human disease [1]. The disease is preceded by a progressive pancreatic inflammatory infiltrate called insulitis, constituted mainly of T cells [2], suggesting a primary role of these cells in diabetes onset. This role is also supported by several studies showing that (1) in vivo treatment with anti-CD4 prevents spontaneous T1D; (2) splenic CD4^+^ and CD8^+^ T cells from diabetic NOD mice can transfer the disease and; (3) neonatal thymectomy prevents T1D onset [3].

T cells precursors develop within the thymus, where they interact with different components of thymic microenvironment during the migratory process that parallels T cell differentiation [4]. Several disorders were identified in the NOD thymus, such as changes in the arrangement of cortical and medullary epithelial cells network, an increase in the deposition of extracellular matrix molecules (ECM) such as fibronectin and laminin and the presence of giant perivascular spaces (PVS), which are up to ten times larger than in normal animals. Giant PVS are filled with mature single-positive (SP) CD4^+^, CD8^+^ [5] and FoxP3^+^ regulatory T cells [6]. These cells are reported to present a defect in the membrane expression of VLA-5 (very late antigen-5, the α5β1 integrin), a fibronectin receptor, suggesting the involvement of this molecule in thymocyte accumulation within the organ [6,7,8]. Conversely, the formation of giant PVS in the NOD thymus seems to result from a progressive accumulation of mature T cells, as in vivo experiments showed similar rates of fluorescein isothiocyanate (FITC)-stained recent thymic emigrants in NOD peripheral lymphoid organs when compared with controls [7], indicating that other cell migration-related molecules could be involved in this process.

Sphingosine-1-phosphate (S1P) is a bioactive sphingolipid, produced mainly by endothelial cells [9], pericytes [10] and erythrocytes [11]. When binding to one of its five receptors (S1P1–5), S1P participates in various cellular processes, such as proliferation, adhesion, death and migration [12,13]. S1P1, S1P2 and S1P3 are ubiquitously expressed, while S1P4 and S1P5 expression is restricted to some cellular types [13]. S1P1 is widely expressed, mainly in endothelial cells, brain, heart and immune cells [14,15]. S1P1 signaling in T cells suppresses proliferation and cytokine production and regulates cell migration [9,16], promoting chemotaxis or fugetaxis depending on the S1P concentration [17]. Accordingly, several studies demonstrate the essential role of S1P and S1P1 in cell migration and, mainly, in the egress of T cells from the thymus to peripheral lymphoid organs. Indeed, S1P1 knockout progenitor T cells are able to enter the thymus and differentiate normally; however, mature T cells cannot leave the organ and are not found in blood and peripheral lymphoid organs [18]. In addition, CD4^+^CD8^+^ double-positive (DP) thymocytes present lower S1P1 membrane expression levels compared with mature CD4^+^ or CD8^+^ SP thymocytes, which present increased migration response towards S1P. Thymocytes increase S1P1 expression during differentiation and leave the organ following a S1P gradient, which is more concentrated in the blood than in the lymphoid organs [12]. Thus, the S1P1 works as a major receptor driving the output of lymphocytes from lymphoid organs under stimulation followed by its internalization, which prevents a constitutive signaling in an environment with high levels of S1P [19]. Biosynthesis of S1P is regulated by sphingosine-kinases and S1P-phosphatases, besides irreversibly degradation by S1P lyase that maintains the gradient of S1P between the compartments. Therefore, high concentrations of S1P in the blood and lymph together with low concentrations within lymphoid organs sustain a constant flow of lymphocytes from lymphoid organs [20].

There is no evidence of the putative role of S1P1 and its physiological ligand, S1P, in the NOD thymus, particularly in the events that precede the onset of T1D, and how this receptor impacts T cell migration and accumulation within the NOD thymus. To address these issues, we investigated herein S1P1 expression and its functional role in thymocyte migration in pre-diabetic NOD mice. We found that NOD mouse thymocytes have decreased expression of S1P1, mainly within mature CD49e^−^ (α5 integrin chain) cells. Functional migration assays revealed that NOD CD62L^hi^CD49e^−^ thymocytes migrate less towards different concentrations of S1P, differing from the CD62L^hi^CD49e^+^ cells that migrate more than controls under the same conditions. Our data indicate that S1P1 is involved in the abnormal NOD thymocyte migration, possibly being associated with the lower VLA-5 expression in NOD thymocytes.

## 2. Results

### 2.1. Decreased S1P1 Expression in NOD Thymocytes

**Figure 1:**
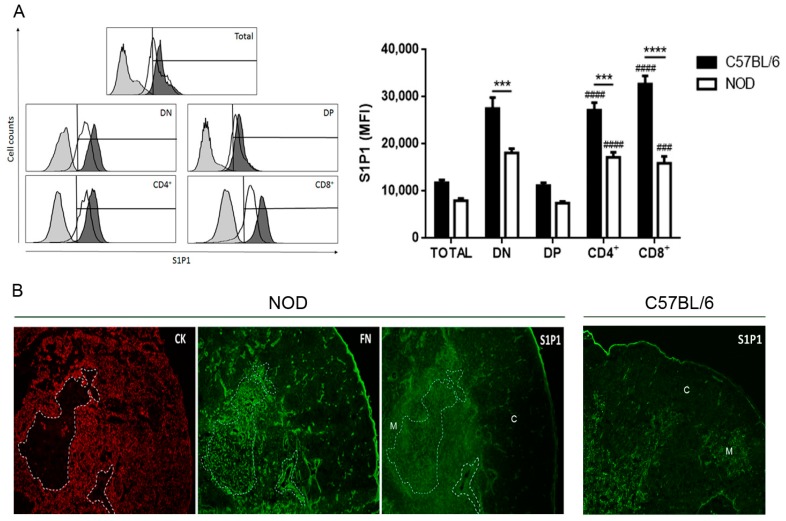

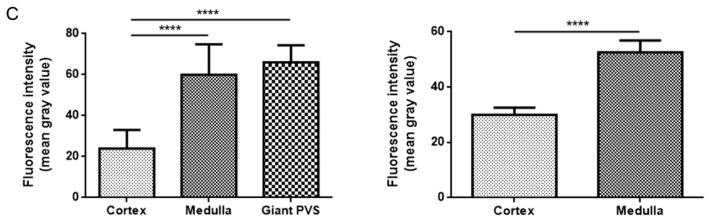
Decreased expression of sphingosine-1-phosphate (S1P1) in non-obese diabetic (NOD) mouse thymocytes. Panel (**A**) shows the S1P1 expression in CD4/CD8-defined thymocyte subpopulations in C57BL/6 (black bars) and NOD (white bars) mice, analyzed by flow cytometry. The histograms show the S1P1 representative staining in NOD (white curve) and C57BL/6 (black curve) thymocyte subpopulations. Grey curves represent the negative staining control for C57BL/6 and the region marks the positive staining. Total = total thymocytes; DN = CD4^−^CD8^−^ double-negative; DP = CD4^+^CD8^+^ double-positive; CD4^+^ = CD4^+^CD8^−^ single-positive; CD8^+^ = CD4^−^CD8^+^ single-positive; MFI = median fluorescence intensity. Results are expressed as mean ± SEM and were analyzed by 2-way ANOVA followed by Tukey’s post-test. Differences were considered statistically significant when *** or ^###^ *p* < 0.001; **** or ^####^ *p* < 0.0001. Asterisks represent statistical significance between C57BL/6 and NOD subpopulations; hash marks represent statistical significance between DP and SP subpopulations in the same mouse strain, after evaluating 3 C57BL/6 and 4 NOD thymi. (**B**) immunohistochemistry showing the S1P1, fibronectin (FN) and cytokeratin (CK) expression profile in NOD and S1P1 expression in C57BL/6 thymus sections. The white dashed lines delimit the giant PVS. C = cortical region; M = medullary region. Three thymi per group were evaluated, with 1 cryosection being analyzed. Original magnification, ×100. (**C**) S1P1 fluorescence intensity in the cortex, medulla and giant PVS of NOD (left graph) and in the cortex and medulla of C57BL/6 (right graph) mice, represented by the mean grey value. **** *p* < 0.0001.

We first analyzed S1P1 expression in NOD mouse thymocytes. As expected and observed by others [12], we observed by flow cytometry that SP thymocytes express higher levels of S1P1 than DP cells in control C57BL/6 mice (Figure 1A). The same kind of difference, between DP and SP subpopulations, was seen in NOD mouse thymocytes. Nevertheless, in this case the expression of S1P1 was lower in CD4^+^ and CD8^+^ SP cells when compared with the correlated subpopulations of C57BL/6 mice (Figure 1A). The reduced expression of S1P1 was more evident in CD8^+^ cells (51.3% of decrease) than in CD4^+^ cells (36.7%). In agreement with flow cytometry results, immunohistochemistry revealed a high expression level of S1P1 in the medullary region of C57BL/6 (Figure 1B,C, right panel) and NOD mouse thymi, as well as within NOD giant PVS (Figure 1B,C, left panel), when comparing with the cortical region. Yet, no differences were detected when comparing the S1P1 immunostaining density in the medulla with the values seen in giant PVS (Figure 1C, left panel).

We also investigated whether the mRNA S1P1 expression correlated with the diminished protein expression in NOD thymocytes. Despite the decreased expression of the receptor when compared with C57BL/6 mice, we found a higher S1P1 mRNA expression in NOD total and CD8^+^ SP thymocytes (Figure S1), suggesting that the impairment in the receptor expression occurs only at protein level and may lead to a positive transcriptional regulation of the S1P1 gene.

**Figure 2:**
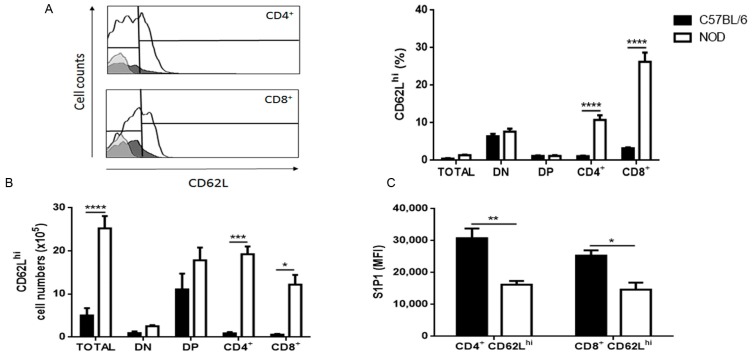

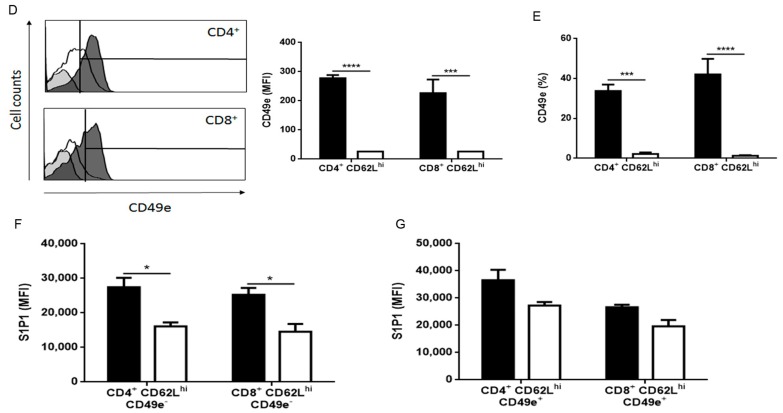
NOD CD62L^hi^ mature thymocytes have decreased expression of S1P1. The graphs show CD62L, S1P1 and CD49e expression in CD4/CD8-defined thymocyte subpopulations in C57BL/6 (black bars) and NOD (white bars) mice, analyzed by flow cytometry. (**A**) CD62L relative cell numbers (%); (**B**) CD62L absolute cell numbers; (**C**) S1P1 expression in CD4^+^CD62L^hi^ and CD8^+^CD62L^hi^ subpopulations; (**D**) CD49e expression in CD4^+^CD62L^hi^ and CD8^+^CD62L^hi^ subpopulations, (**E**) percentage of CD4^+^ and CD8^+^CD62L^hi^ cells expressing CD49e; (**F**) S1P1 expression in CD4^+^CD62L^hi^CD49e^−^ and CD8^+^CD62L^hi^CD49e^−^ cells; and (**G**) S1P1 expression in CD4^+^CD62L^hi^CD49e^+^ and CD8^+^CD62L^hi^CD49e^+^ cells. Histograms show the CD62L (**A**) and CD49e (**D**) staining in NOD (white curve) and C57BL/6 (black curve) CD4^+^ and CD8^+^ SP thymocytes. The grey curves represent the negative staining control for C57BL/6. The region marks the positive staining in the case of CD49e and the neg/lo vs. hi (negative/ low vs. high) populations in the CD62L histograms. Total = total thymocytes; DN = CD4^−^CD8^−^ double-negative; DP = CD4^+^CD8^+^ double-positive; CD4^+^ = CD4^+^CD8^−^ single-positive; CD8^+^ = CD4^−^CD8^+^ single-positive; MFI = median fluorescence intensity; % = relative cell numbers. Results are expressed as mean ± SEM and were analyzed by 2-way ANOVA followed by Tukey’s post-test. Differences were considered statistically significant when * *p* < 0.05; ** *p* < 0.01; *** *p* < 0.001; **** *p* < 0.0001. *n* = 3 C57BL/6; *n* = 4 NOD.

We next evaluated the expression of S1P1 in more mature SP thymocytes. We observed an important increase in the percentage and absolute numbers of CD62L^hi^ cells in NOD mouse thymus (Figure 2A,B), in keeping with the accumulation of mature thymocytes within giant PVS [6]. Interestingly, both SP CD4^+^CD62L^hi^ and CD8^+^CD62L^hi^ NOD thymocytes presented a decrease in S1P1 expression (Figure 2C) as well as an important decrease in CD49e expression (Figure 2D,E), when compared with controls. In addition, those NOD SP CD4^+^CD62L^hi^ and CD8^+^CD62L^hi^ cells that did not express CD49e exhibited decreased expression of S1P1 when compared with controls, whereas CD49e^+^CD4^+^CD62L^hi^ and CD49e^+^CD8^+^CD62L^hi^ cells had no significant differences in S1P1 expression densities (Figure 2F,G).

Interestingly, when we analyzed the less mature SP CD4^+^CD62L^neg/lo^ and CD8^+^CD62L^neg/lo^ subpopulations, we found that both NOD CD49e^−^ and CD49e^+^ cells had lower expression of S1P1 (Figure S2A,B), suggesting that maturation of the VLA-5^+^ cells from CD62L^neg/lo^ to the CD62L^hi^ phenotype could be observed by the upregulation in S1P1 expression, although this profile was observed only in NOD CD4^+^ SP cells (Figure S2C,D).

We also analyzed S1P1 expression in mice at 4 weeks of age, an age-point corresponding with the beginning of many alterations found in NOD mice, including the formation of enlarged PVSs [21,22,23,24,25]. Curiously, we found no differences in any of the populations investigated (Figures S3A–F and S4A–F), suggesting that the decreased expression of S1P1 appears late in NOD mouse life and after the VLA-5 defect. Together, these results indicate that the reduced expression of S1P1, along with the VLA-5 defect in mature SP thymocyte subpopulations in NOD mice, could modulate the migratory capacity of thymocytes and contribute to the accumulation of these cells in giant PVS.

### 2.2. Decreased Expression of S1P Lyase 1 in NOD Thymocytes

**Figure 3:**
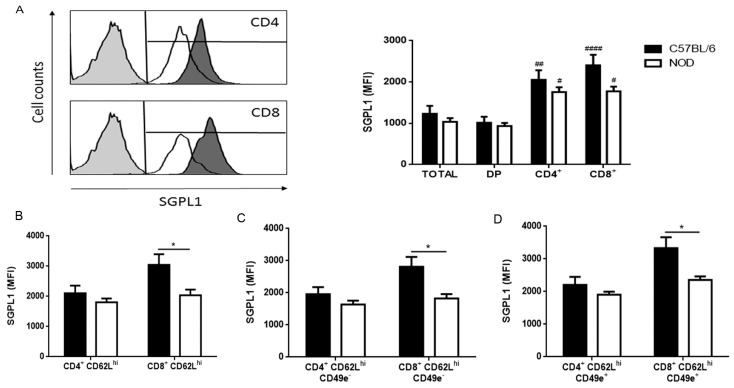

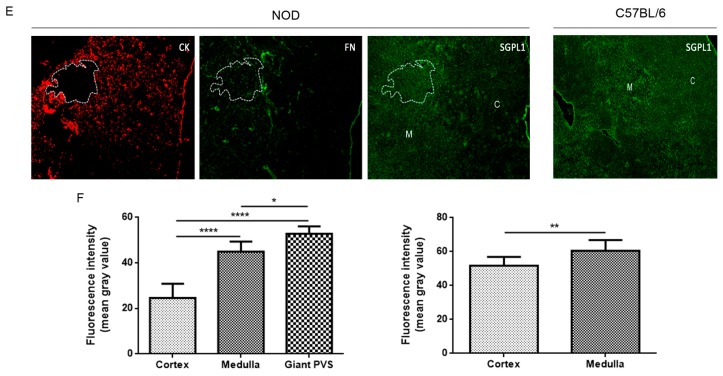
NOD thymocytes have reduced expression of S1P lyase 1 (SGPL1). The graphs show the SGPL1 expression in CD4/CD8-defined thymocyte subpopulations in C57BL/6 (black bars) and NOD (white bars) mice, analyzed by flow cytometry. (**A**) SGPL1 in total thymocytes, as well as CD4^+^CD8^+^ double-positive, CD4^+^ and CD8^+^ SP subpopulations. Histograms show the SGPL1 staining in NOD (white curve) and C57BL/6 (black curve) CD4^+^ and CD8^+^ SP thymocytes. The grey curves represent the negative staining control; (**B**) SGPL1 in mature CD4^+^CD62L^hi^ and CD8^+^CD62L^hi^ SP thymocyte subpopulations; (**C**) SGPL1 expression in CD49e^−^CD4^+^CD62L^hi^ and CD49e^−^CD8^+^CD62L^hi^ SP thymocytes; (**D**) SGPL1 expression in CD49e^+^CD4^+^CD62L^hi^ and CD49e^+^CD8^+^CD62L^hi^ SP thymocytes; (**E**) SGPL1, fibronectin (FN) and cytokeratin (CK) expression profile in NOD and SGPL1 expression in C57BL/6 thymus sections. The white dashed lines delimit the giant PVS. C = cortical region; M = medullary region. *n* = 3 thymi per group. Original magnification, ×100; and (**F**) SGPL1 fluorescence intensity quantification in the cortex, medulla and giant PVS of NOD (left graph) and in the cortex and medulla of C57BL/6 (right graph) mice, represented by the mean grey value. Total = total thymocytes; DN = CD4^−^CD8^−^ double-negative; DP = CD4^+^CD8^+^ double-positive; CD4^+^ = CD4^+^CD8^−^ single-positive; CD8^+^ = CD4^−^CD8^+^ single-positive; MFI = median fluorescence intensity. Results are expressed as mean ± SEM and were analyzed by 2-way ANOVA followed by Tukey’s post-test. Differences were considered statistically significant when * or ^#^ *p* < 0.05; ** or ^##^ *p* < 0.01; **** or ^####^ *p* < 0.0001. Asterisks represent a statistically significant difference between C57BL/6 and NOD subpopulations; hash marks represent a statistically significant difference between DP and SP subpopulations in the same mouse strain. *n* = 8 C57BL/6; *n* = 8 NOD.

S1P lyase 1 (SGPL1) maintains constant S1P concentrations in tissues by irreversible degradation of S1P [26]. In the thymus, SGPL1 expression in the medullary region is related to low S1P concentrations around the vessels, allowing up-regulation of S1P1 and egress of mature thymocytes [27,28]. Inhibition of SGPL1 leads to the accumulation of mature thymocytes in the thymus and giant PVS formation, secondary to S1P1 internalization [28,29]. We observed higher SGPL1 expression densities in SP CD4^+^ and CD8^+^ thymocytes when compared with DP cells of both C57BL/6 and NOD mice (Figure 3A), similar to when we compared CD49e^−^ and CD49e^+^ cells (Figure S5A,B). Although differences between subpopulations were observed in both mouse strains, they were less important concerning NOD mouse thymocytes. No differences in the relative numbers of cells expressing SGPL1 were observed (Figure S6A–H). Interestingly, when we analyzed the CD62L^hi^ subpopulations, NOD SP CD8^+^CD62^hi^, CD8^+^CD62L^hi^CD49e^−^ and CD8^+^CD62L^hi^CD49e^+^ thymocytes presented lower SGPL1 expression in comparison with C57BL/6 counterparts (Figure 3B–D). The same was observed for NOD SP CD8^+^CD62L^neg/lo^CD49e^+^, while a relevant, but not significant reduction was seen in SP CD8^+^CD62L^neg/lo^CD49e^−^ cells (Figures S5C,D). In agreement with the increase of SGPL1 expression in SP cells by flow cytometry, we observed by immunohistochemistry a higher SGPL1 deposition in the medullary region of both C57BL/6 (Figure 3E,F, right panel) and NOD (Figure 3E,F, left panel) thymi. Accordingly, the SGPL1 reduction in mature NOD thymocytes could result in less degradation and consequent accumulation of S1P around PVS leading to S1P1 internalization and thymocyte accumulation.

### 2.3. CD49e-Negative NOD Mouse Thymocytes Have Impaired S1P-Driven Migratory Response

**Figure 4:**
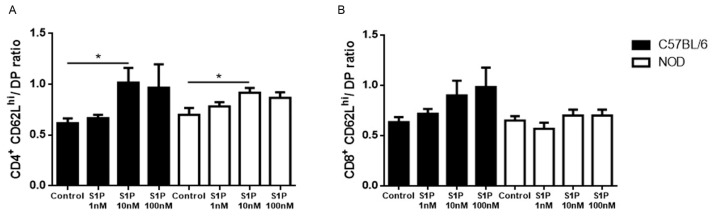
S1P induces NOD thymocyte migration. Total thymocytes of C57BL/6 (black bars) and NOD (white bars) mice were added to the Transwell insert and allowed to respond to 1, 10 or 100 nM of S1P in the lower chamber. Single-positive (**A**) CD4^+^CD62L^hi^; and (**B**) CD8^+^CD62L^hi^ and double-positive (DP) percentages of input were determined by flow cytometry. Results are shown as the ratio of the percentages of input (single-positive to double-positive thymocytes). Results are expressed as mean ± SEM and were analyzed by Student’s *t* test. Differences were considered statistically significant when * *p* < 0.05. *n* = 6 C57BL/6; *n* = 6 NOD.

We investigated the functional role of S1P/S1P1 interactions in NOD thymocyte subpopulations in Transwell^®^ (Corning Costar, Cambridge, MA, USA) migration assays. We observed that both the C57BL/6 and NOD SP CD4^+^CD62L^hi^ subpopulation migrated towards 10 nM of S1P (Figure 4A), whereas a discrete, but not significant, migratory response was seen only in C57BL/6 SP CD8^+^CD62L^hi^ cells towards both 10 and 100 nM S1P concentrations (Figure 4B). No chemotactic response was observed when the C57BL/6 or NOD SP CD62L^neg/lo^ subpopulations were stimulated with the same concentrations of S1P and, in some cases, the migration was even lower than in the control (Figure S7A,B).

**Figure 5:**
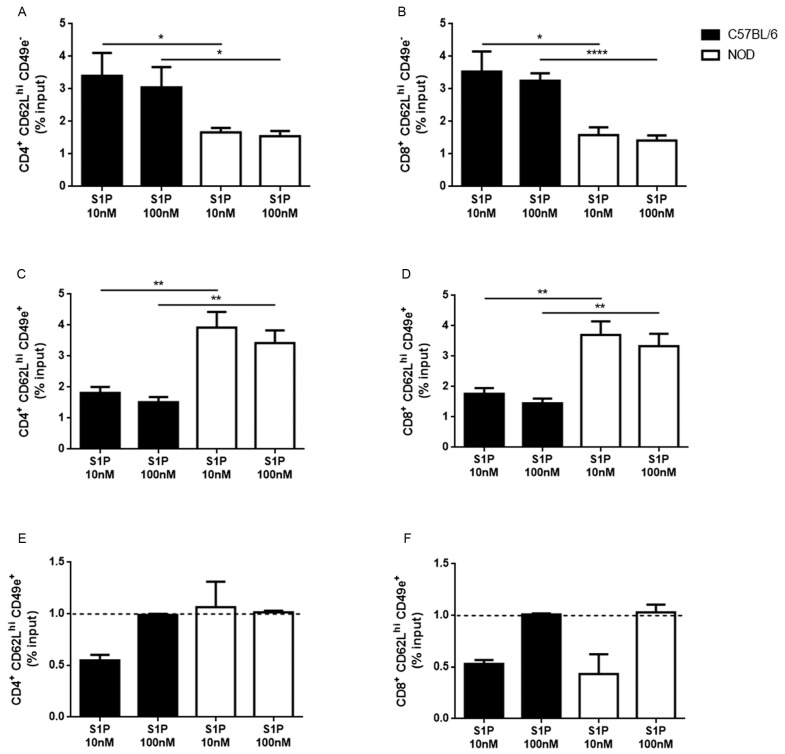
NOD CD49e^−^ and CD49e^+^ thymocytes present different migration patterns under S1P stimulation. Total thymocytes of C57BL/6 (black bars) and NOD (white bars) mice were added to the Transwell insert and were allowed to migrate toward 10 or 100 nM of S1P present in the lower chamber. Single-positive (**A**) CD4^+^CD62L^hi^CD49e^−^; (**B**) CD8^+^CD62L^hi^CD49e^−^; (**C**) CD4^+^CD62L^hi^CD49e^+^; and (**D**) CD8^+^CD62L^hi^CD49e^+^ percentages of input were determined by flow cytometry, (**E**) percentages of input of CD4^+^CD62L^hi^CD49e^+^; and (**F**) CD8^+^CD62L^hi^CD49e^+^ after CD49 blockade, compared with isotype control-treated cells (dashed black lines = 1). Results are expressed as mean ± SEM and were analyzed by Student’s *t* test. Differences were considered statistically significant when * *p* < 0.05; ** *p* < 0.01; **** *p* < 0.0001. *n* = 6 C57BL/6 and 6 NOD (**A**–**D**) and *n* = 4 C57BL/6 and 4 NOD (**E**,**F**).

We also investigated whether the differences in S1P1 expression between CD49e^+^ and CD49e^−^ cells correlated with the changes in migratory response towards S1P. We found that NOD SP CD62L^hi^CD49e^−^ cells had decreased migratory response toward 10 and 100 nM of S1P when compared with controls (Figure 5A,B). In contrast, NOD SP CD62L^hi^CD49e^+^ showed higher migratory response towards the same S1P concentrations (Figure 5C,D). Regarding CD62L^neg/lo^ cells, there was no difference in migratory responses between NOD and C57BL/6 SP CD4^+^CD62L^neg/lo^CD49e^−^ cells, but NOD SP CD8^+^CD62L^neg/lo^CD49e^−^ cells displayed lower migratory capacity towards 10 nM of S1P (Figure S7C,D). For CD49e^+^ cells, only the NOD SP CD4^+^CD62L^neg/lo^ subpopulation presented higher migratory capacity towards 10 and 100 nM of S1P (Figure S7E,F). Interestingly, when we treated CD62L^hi^CD49e^+^ cells with a specific CD49e blocker antibody, we observed a reduced migratory capacity of C57BL/6 SP CD4^+^ and CD8^+^CD62L^hi^CD49e^+^ and NOD SP CD8^+^CD62L^hi^CD49e^+^ cells toward 10 nM of S1P, while migration toward 100 nM of S1P remained unaltered (Figure 5E,F). Together, these results show that the membrane levels of VLA-5 (ascertained by CD49e expression) can influence the cellular migratory response to S1P in the thymus, particularly in the NOD mouse.

## 3. Discussion

S1P1 is the main receptor involved in thymocyte egress to the periphery [30]. Here, we show that in the thymus of the NOD mouse there is an abnormality in the expression and function of S1P1. NOD thymocytes have a decreased expression of S1P1 when compared with C57BL/6 thymocytes, and this is mainly observed in CD4^+^ and CD8^+^ SP subpopulations, particularly in the more mature SP CD4^+^CD62L^hi^ and SP CD8^+^CD62L^hi^ subpopulations, which under normal conditions, are those ready to leave the organ [30]. In this respect, the NOD thymus also exhibits higher relative and absolute numbers of CD62L^hi^ cells. These mature thymocytes also show decreased expression of CD49e, consistent with the accumulation of mature thymocytes in giant PVS [5,6,7]. Interestingly, we found that the diminished expression of S1P1 occurs in NOD mature (SP CD4^+^CD62L^hi^ and CD8^+^CD62L^hi^) CD49e^−^ thymocytes, but not in CD49e^+^ cells. We observed differences in the percentage of SP CD8^+^CD62L^hi^ cells that express S1P1, as well as SP CD8^+^CD62L^hi^CD49e^−^ and CD8^+^CD62L^hi^CD49e^+^, when comparing C57BL/6 with NOD (Figure S8A–F); the main differences were observed in the density of receptor expression ascertained by the median of fluorescence intensity measurements. These data suggest that the expression of S1P1 and the integrin VLA-5 is somehow related, resulting in the involvement of both molecules in the accumulation of mature thymocytes in the NOD thymus. This hypothesis is supported by several studies showing that the signaling mediated by S1P1 modulates integrin activation and localization. Pre-treatment of endothelial cells with S1P prevented monocyte adhesion through VLA-5 and αvβ3 integrins expressed on the endothelial apical surface. The treatment changed the localization of the integrins to the basal surface through S1P1 signaling and activation of proto-oncogene tyrosine-protein kinase Src family proteins, PI3K (phosphoinositide 3-kinase) and Rac (Ras-related C3 botulinum toxin substrate) [31]. S1P activates the integrin αvβ3 in the endothelial cell lamellipodial region and promotes cell migration through vitronectin substrates. The S1P signaling via S1P1/Gi/Rho GTPases induces integrin association with cytoskeleton proteins and the combination of αv and β3 subunits [32]. Furthermore, it has been shown that S1P/S1P1 interactions enhance the CXCL12-mediated myeloma cell adhesion to fibronectin through VLA-4 (the α4β1 integrin) and VLA-4-mediated transendothelial migration [33].

We also investigated the mRNA expression of S1P1 in NOD versus C57BL/6 thymocytes. Curiously, the S1P1 mRNA expression was higher in NOD mice than in C57BL/6, both in total and mature CD8^+^ SP cells. These data suggest that the decreased expression of the receptor occurs only at the protein level, which may, in turn, result in a positive transcriptional regulation of the *S1P1* gene. One explanation could be the desensitization of G protein-coupled receptors that occurs rapidly after agonist exposure and a decrease in the membrane expression of the receptors that can be observed after prolonged agonist exposure [34]. Moreover, high concentrations of S1P were able to induce internalization, followed by ubiquitination and degradation of S1P1 [35]. Thymocytes are highly sensitive to S1P and internalize S1P1 after incubation with 1 nM of this lipid [36]. This internalization in mature thymocytes can prevent cells from exiting the organ, and increased S1P concentrations in thymic medullary region (mainly around the PVS) likely caused by SGPL1 inhibition, can lead to the formation of giant PVS in normal mice [28]. In this study, we did not observe a breakdown of the S1P gradient in NOD mice when comparing S1P contents between the thymus and serum, which were similar to C57BL/6 controls (Figure S9). Nevertheless, we cannot rule out the hypothesis of dysregulation in S1P concentration in the NOD thymus microenvironment, such as an increase around and within the giant PVS. Higher S1P concentrations could lead to a decrease in the expression of S1P1 in CD49e^−^ cells that remain for longer periods in these structures. We observed lower SGPL1 expression in NOD mature SP CD8^+^CD62L^hi^ thymocytes as compared with controls. Downregulation or inhibition of this enzyme causes lymphopenia through the disruption of S1P gradients inside the thymus [29]. Although dendritic cells (DCs) present in the medullary and cortico-medullary regions seem to be the main reason for the low levels of S1P, the disruption of SGPL1 in thymocytes was also related to increased thymus and plasma levels of S1P, the decrease of S1P1 on the mature SP cell surface and mature thymocyte retention in the thymus [37]. However, other events that involve post-translational regulation of the receptor expression cannot be discarded.

We observed that S1P1 decreased expression correlated with a lower migratory response of NOD thymocytes towards S1P. Additionally, S1P-driven migration of NOD SP CD62L^hi^CD49e^−^ cells was lower than in the controls, although migration of NOD mature SP CD62L^hi^CD49e^+^ thymocytes was higher when induced by the same S1P concentrations. It is interesting to emphasize that CD49e^−^ mature thymocytes migrate more towards S1P than CD49e^+^ mature cells in C57BL/6 mice, but not in NOD mice. In this case, CD49e^+^ mature cells were more responsive to S1P, suggesting that the CD49e expression can influence the chemotactic responses towards S1P in the NOD thymus in a different way.

Finally, we observed a decreased migratory capacity of C57BL/6 SP CD4^+^ and CD8^+^CD62L^hi^CD49e^+^ towards 10 nM of S1P after CD49e blockade, suggesting that this integrin can indirectly regulate S1P-induced thymocyte migration. In contrast, inhibition of migration was observed for NOD CD8^+^CD62L^hi^CD49e^+^ thymocytes, suggesting that in this subpopulation other molecules can also be involved. Distinct migratory responses between CD4^+^ and CD8^+^ SP thymocytes from NOD mice were described, for example, towards laminin. In this case, laminin receptor VLA-6 expression is augmented in both CD4^+^ and CD8^+^ SP thymocytes, but migration is enhanced only for CD4 SP cells [6]. Furthermore, our group showed that S1P induces chemotaxis and chemokinesis of T-cell acute lymphoblastic leukemia (T-ALL) blasts at 100 nM. By contrast, high concentrations of S1P (1000, 5000 and 10,000 nM) induce fugetaxis (or chemorepulsion) of the same cells [17]. It is thus conceivable that different thymocyte subpopulations present different sensitivity in migratory behavior under S1P stimulation, depending on the concentration and receptor expression, and that VLA-5 expression could differentially influence cell response in these conditions. Indeed, we cannot exclude the participation of other interactions since S1P/S1P1 can alter cellular responsiveness through other molecules, besides integrin activation and chemokine response, as mentioned above. Together, our results provide evidence that the expression and migratory defects of NOD thymocytes are part of a complex mechanism that comprises hypo and hyper responsiveness to certain stimuli. This fits the multivectorial model for intrathymic T-cell migration we have previously proposed [6], in which the direction and velocity of thymocyte migration result from the balance of interactions mediated by different vectors, such as ECM proteins, cytokines and chemokines. Accordingly, the alterations seen in NOD mouse thymocytes likely alter the resultant vector driving oriented thymocyte movement within the thymus, modifying the overall thymocyte migratory behavior, which should be considered in the context of T1D development in NOD mice.

## 4. Materials and Methods

### 4.1. Animals

Female C57BL/6 and NOD mice aged 9–12 weeks (pre-diabetic) or 4 weeks (when indicated) were obtained from the Institute of Science and Technology in Biomodels (ICTB, Fiocruz, Rio de Janeiro, Brazil) and the Necker Hospital (Paris, France) and were maintained under specific-pathogen free (SPF) conditions. Experimental procedures were approved by the Fiocruz ethical committee on animal use (L-024/2015; June 2015), according to the rules defined by Brazilian and European legislations. 

### 4.2. Antibodies and Chemicals

Rabbit anti-S1P1 (catalog number PA1-1040) and S1P1 Synthetic Immunizing Peptide (PEP-220) were obtained from Thermo Scientific (Rockford, IL, USA). Rabbit anti-SGPL1 (bs-4188R) was purchased from Bioss Antibodies (Boston, MA, USA). S1P and rabbit anti-FN (F3648) were obtained from Sigma Aldrich (St. Louis, MO, USA). Fluorochrome-labeled rat monoclonal antibodies directed against mouse proteins, including anti-CD4 APC, anti-CD8 PerCP, anti-CD49e PE, unrelated IgG2A PE and anti-CD4 APC-Cy7, were purchased from BD Biosciences (San Jose, CA, USA), as well as purified rat anti-CD49e and its respective isotype control. Rat anti-CD62L Pacific Blue and the respective isotype control were obtained from BioLegend (San Diego, CA, USA). Purified rabbit anti-CK polyclonal antibody was obtained from DAKO Co. (Santa Clara, CA, USA). Rat anti-CD8 PerCP-Cy5.5, goat anti-rabbit IgG Alexa Fluor^®^488 and goat anti-rabbit IgG Alexa Fluor^®^ 546 were obtained from Molecular Probes (Eugene, OR, USA).

### 4.3. Flow Cytometry

After thymus removal, thymocyte suspension was prepared in a tissue homogenizer with 1 mL of PBS (Sigma Aldrich). One million cells were stained using the BD Cytofix/CytopermTM Fixation/Permeabilization Kit (BD Biosciences) for S1P1, followed by staining for CD49e, CD62L, CD4 and CD8. Cells were then evaluated by flow cytometry using a FACSCanto II device (BD Biosciences).

### 4.4. Real-Time Quantitative Polymerase Chain Reaction (Quantitative RT-PCR)

Thymus RNA was extracted using a combination of TRIzol^®^ (Invitrogen, Carlsbad, CA, USA) and RNeasy^®^ mini kit (Qiagen, Austin, TX, USA): total thymi were lysed in 1 mL TRIzol^®^ with the aid of a pipette. Chloroform was then added to the lysate, and the organic and aqueous phases were separated by centrifugation at 10,000× *g* for 18 min, at 4 °C. From the aqueous phase, after the precipitation with ethanol, RNA was extracted using the RNeasy^®^ mini kit and suspended in Ultrapure Nuclease-Free Water (USB Corporation, Cleveland, OH, USA). cDNA synthesis was performed with 2 μg of RNA with Super Script II RT (Invitrogen, Carlsbad, CA, USA). For SP CD4^+^ and CD8^+^ subpopulations, a pool of thymocytes from 4 thymi was suspended in 1 mL of RPMI (Roswell Park Memorial Institute medium) 1640 10% FBS (fetal bovine serum) (Cultilab, Campinas, Brazil) and stained with anti-CD4 and anti-CD8 antibodies for 30 min at 4 °C, followed by cell sorting in a FACS Aria device (BD Biosciences). After sorting, RNA was extracted from SP CD4^+^ and CD8^+^ cells using the same protocol described above. cDNA synthesis was performed with 100 ng of RNA. Quantitative RT-PCR of total thymus and SP subpopulations was performed with Syber^®^ Green PCR Master Mix (Applied Biosystems, Forster City, CA, USA) on a Step One Plus System (Applied Biosystems). PCR cycling conditions were as follows: a first step at 95 °C for 20 s, followed by 40 cycles at 95 °C for 3 s and 62 °C for 30 s and generation of melting curves for primer specificity analysis. Gene expression was calculated in the Expression Suite Software (version 1.1., Life Technologies, Carlsbad, CA, USA) using the comparative *C*t method (ΔΔ*C*t) with the threshold set at 0.02. Statistical analyses were conducted using the Δ*C*t values. Gene expression was reported as fold change (2^−ΔΔ*C*t^), in relation to samples from C57BL/6 control mice, used as calibrators. HPRT and GAPDH genes were used as reference genes and their constitutive expression was validated using the same software. The following primers were used: 200 nM S1P1 forward GTGTAGACCCAGAGTCCTGCG; 200 nM S1P1 reverse AGCTTTTCCTTGGCTGGAGAG (Sigma Aldrich); 300 nM HPRT forward TCCCAGCGTCGTGATTAGCGATG; 300 nM HPRT reverse GGCCACAATGTGATGGCCTCCC (Invitrogen, Carlsbad, CA, USA); 300 nM GAPDH forward CCACTCACGGCAAATTCAACGGC; 300 nM GAPDH reverse CCACCCTTCAAGTGGGCCCCG (Invitrogen, Carlsbad, CA, USA). Further information about primer standardization can be found in supplementary information, Table S1.

### 4.5. Immunohistochemistry

Thymi were removed from NOD and C57BL/6 mice, embedded in Tissue-Tek O.C.T. Compound (Sakura Finetechnical Co., Tokyo, Japan) and maintained at −80 °C. 5-μm-thick cryostat sections were settled on poly-l-lysine (Sigma Aldrich)-covered glass slides and were acetone-fixed for 10 min at −20 °C. Slides were treated with PBS 1% BSA (bovine serum albumin) for 30 min and incubated with primary antibodies (diluted in PBS for extracellular staining or PBS/BSA 1%/Saponin 0.1% for intracellular staining) overnight at 4 °C. Samples were then submitted to corresponding secondary antibodies for 30 min at room temperature. Immunostained samples were analyzed by an Axio Imager A2 device using the Axio Vision Rel 4.8 software (Zeiss, Oberkochen, Germany). Negative controls, in which the secondary antibody was used alone, did not generate any significant labeling. Selected microscopic fields comprised cortical, medullary and PVS regions of the thymic lobules. The quantitative fluorescence analysis was performed by transforming the specific staining into an eight-bit grey image. The Mean Gray Value was used to quantify S1P1 or SGPL1 expression, using ImageJ software (Rasband, WS ImageJ, NIH, Bethesda, Rockville, MD, USA). For S1P1 expression, we examined 23 cortical, 8 medullary and 11 giant PVS regions for NOD thymus and 33 cortical and 24 medullary regions for C57BL/6. For SGPL1 (Sphingosine-1-phosphate lyase 1) expression, we examined 11 cortical, 7 medullary and 4 giant PVS regions for NOD and 12 cortical and 9 medullary regions, for C57BL/6 thymus.

### 4.6. Transmigration Assays

Thymocyte migratory activity was assessed in Transwell^®^ chambers. Briefly, 5-µm pore size Transwell membranes (Corning Incorporated Costar, Corelle, NY, USA) were blocked with PBS 1 % fatty-acid free BSA for 45 min at 37 °C in a 5 % CO_2_ atmosphere. After blockade, 2 × 10^6^ cells in 100 μL of RPMI 1640 1 % fatty-acid free BSA migration medium were added to the upper chamber, while 600 μL of the migration medium or migration medium containing S1P were added to the bottom chamber. After 3 h of incubation, the cells in the bottom chamber were counted in a Neubauer hematocymometer (BRAND GMBH + CO KG, Wertheim, Germany), followed by labeling with appropriate antibodies and analyzed by flow cytometry. For CD49e blockade assays, cells were pre-incubated with blocking antibody or isotype control for 30 min at 4 °C and then challenged to migrate under the same conditions described above.

### 4.7. S1P Quantitation

Thymi and sera were obtained from NOD and C57BL/6 mice, and the S1P concentration was determined by thin-layer chromatography (TLC) as previously described [38,39].

### 4.8. Statistical Analysis

Results were analyzed using Student’s *t* test or 2-way ANOVA tests, followed by Tukey’s post-test, and using the software GraphPad Prism 6 (version 6.01, GraphPad Software, San Diego, CA, USA). Differences were considered statistically significant when *p* < 0.05 (* or ^#^), *p* < 0.01 (** or ^##^), *p* < 0.001 (*** or ^###^) or *p* < 0.0001 (**** or ^####^).

## Supplementary Materials

Supplementary materials can be found at http://www.mdpi.com/1422-0067/19/5/1446/s1.

## Abbreviations

NOD	Non-Obese Diabetic
VLA	Very Late Antigen
S1P	Sphingosine-1-Phosphate
S1P1	Sphingosine-1-Phosphate receptor 1
T1D	Type I Diabetes
PVS	Perivascular Spaces
ECM	Extracellular Matrix
SGPL1	S1P lyase 1
