# Supplementary material for: Sphingosine-1-Phosphate Receptor 1 Is Involved in Non-Obese Diabetic Mouse Thymocyte Migration Disorders
Source: Int J Mol Sci. 2018 May 12;19(5):1446. doi: 10.3390/ijms19051446 (PMC5983610; doi:10.3390/ijms19051446)
Supplement: Supplementary file 1 [file ijms-19-01446-s001.pdf]

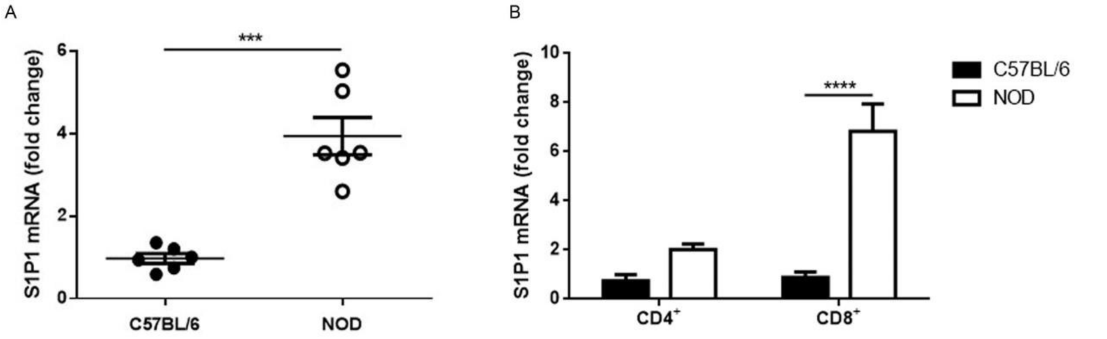


**Figure S1.** NOD thymocytes have increased expression of S1P1 mRNA. (A), the graph shows the mRNA expression of S1P1 in total thymocytes of C57BL/6 (black balls) and NOD (white balls) mice. Panel B depicts the mRNA expression of S1P1 in CD4$^+$ and CD8$^+$ SP thymocyte subpopulations in C57BL/6 (black bars) and NOD (white bars) mice. CD4$^+$ = CD4$^+$CD8$^-$ single-positive; CD8$^+$ = CD4$^-$CD8$^+$ single-positive. The relative quantitation was performed by the comparative Ct method (△△Ct), using C57BL/6 as calibrator (fold change value = 1). Horizontal bars represent the mean ± SEM of six biological replicates for total thymocytes (A) and sixteen biological replicates for SP subpopulations (B). Results were analyzed by Student's t test for total thymus and 2 way ANOVA followed by Tukey post-test for sorted thymocyte subsets. Differences were considered statistically significant when *** $p < 0.001$; **** $p < 0.0001$.

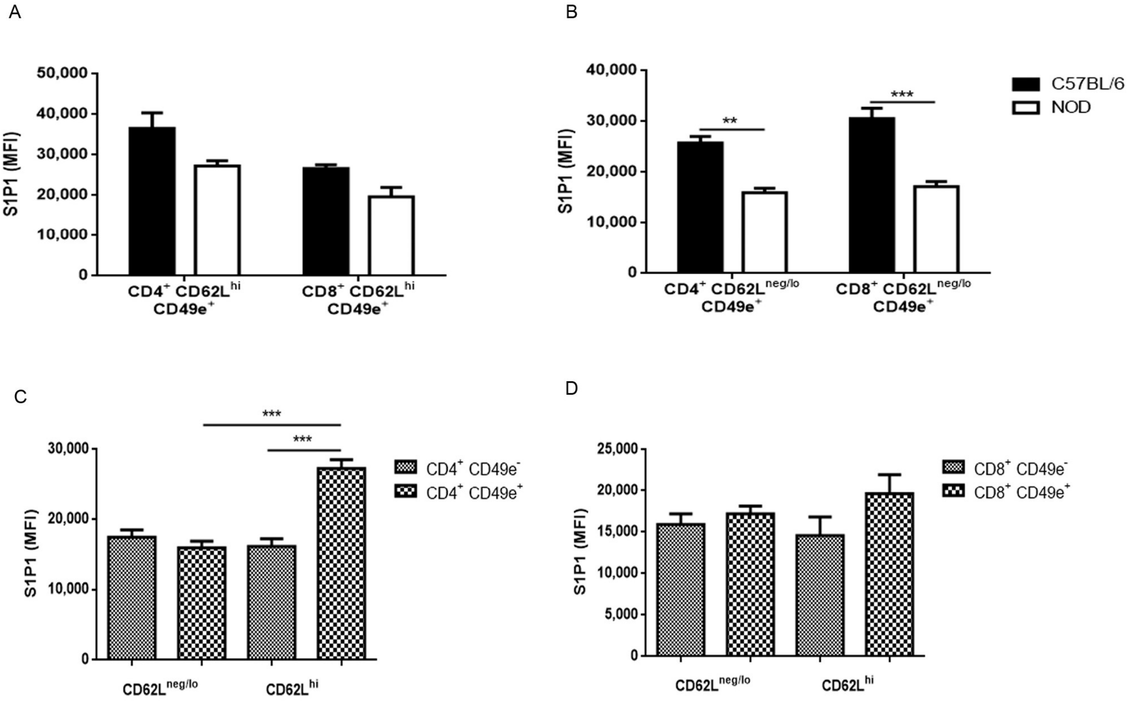


**Figure S2.** NOD CD4$^+$CD49e$^+$ SP cells increase S1P1 expression during differentiation. The panels show S1P1 expression in (A) CD4$^+$ and CD8$^+$ SP CD62L$^{neg/lo}$CD49e$^-$ cells, (B), SP CD4$^+$ and CD8$^+$ CD62L$^{neg/lo}$CD49e$^+$ cells, (C), NOD SP CD4$^+$CD62L$^{neg/lo}$ or CD4$^+$CD62L$^{hi}$, expressing or not CD49e and (D) NOD SP CD8$^+$CD62L$^{neg/lo}$ or CD8$^+$CD62L$^{hi}$ thymocytes expressing or not CD49e, as indicated, analyzed by flow cytometry. CD4$^+$ = CD4$^+$CD8$^-$ single-positive; CD8$^+$ = CD4$^-$CD8$^+$ single-positive; MFI = median fluorescence intensity. Results are expressed as mean ± SEM and were analyzed by 2way ANOVA followed by Tukey post-test or Student's t test. Differences were considered statistically significant when ** $p < 0.01$; *** $p < 0.001$; **** $p < 0.0001$, after evaluating 3 C57BL/6 and 4 NOD thymi.

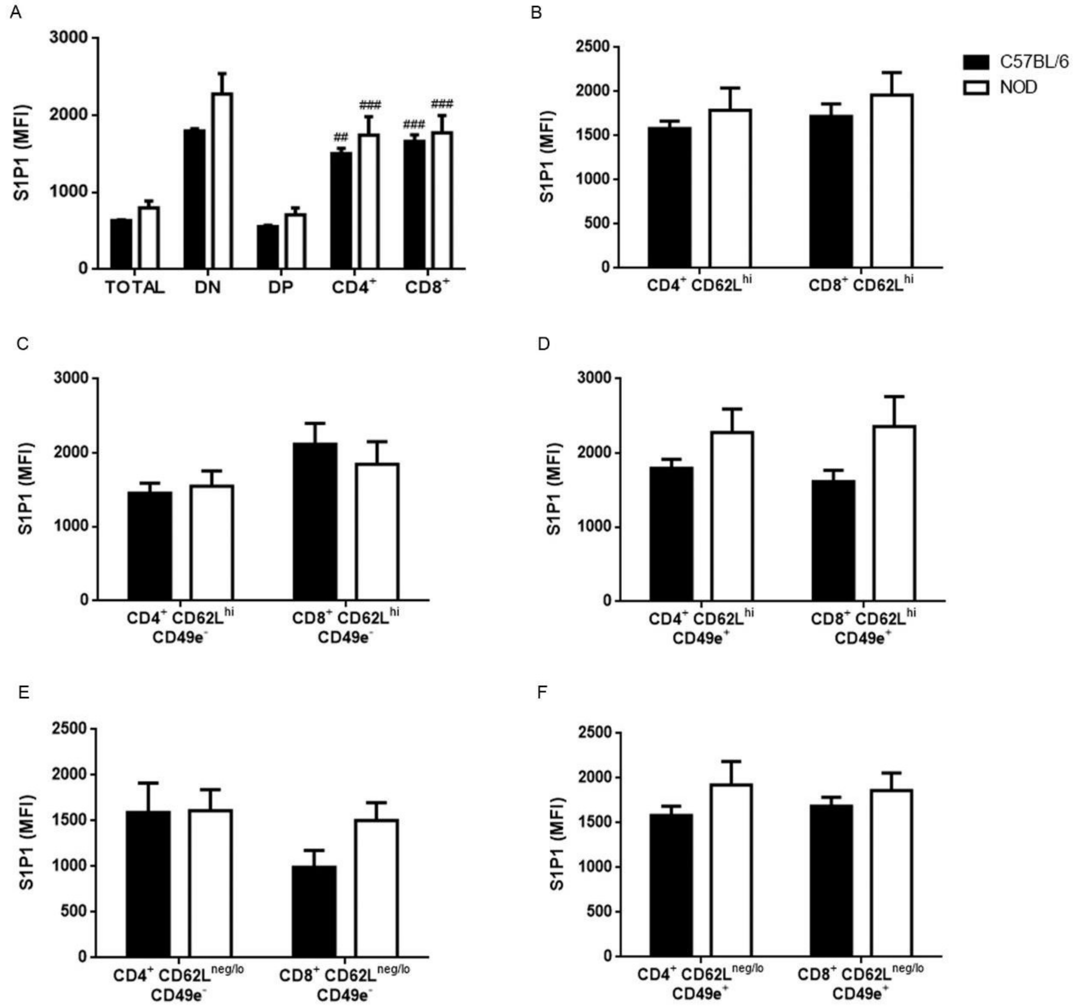


**Figure S3.** Younger NOD mice have no alterations in S1P1 expression. The graphs show S1P1 expression in C57BL/6 (black bars) and NOD (white bars) mice with 4 weeks of age, analyzed by flow cytometry. (A), S1P1 expression on CD4/CD8$^-$defined thymocyte subpopulations, (B), S1P1 expression in CD4$^+$CD62L$^{hi}$ and CD8$^+$CD62L$^{hi}$ subpopulations. (C), S1P1 expression in CD4$^+$CD62L$^{hi}$CD49e$^-$ and CD8$^+$CD62L$^{hi}$CD49e$^-$ cells and (D), S1P1 expression in CD4$^+$CD62L$^{hi}$CD49e$^+$ and CD8$^+$CD62L$^{hi}$CD49e$^+$ cells, (E), S1P1 expression in CD4$^+$CD62L$^{neg/lo}$CD49e$^-$ and CD8$^+$CD62L$^{neg/lo}$CD49e$^-$ cells and (F), S1P1 expression in CD4$^+$CD62L$^{neg/lo}$CD49e$^+$ and CD8$^+$CD62L$^{neg/lo}$CD49e$^+$ cells. Total = total thymocytes; DN = CD4$^-$CD8$^-$ double$^-$ negative; DP = CD4$^+$CD8$^+$ double-positive; CD4$^+$ = CD4$^+$CD8$^-$ single–positive; CD8$^+$ = CD4$^-$CD8$^+$ single-positive; MFI = median fluorescence intensity. Results are expressed as mean ± SEM and were analyzed by 2way ANOVA followed by Tukey post-test. Differences were considered statistically significant when ## $p < 0.01$; ### $p < 0.001$. Hash marks represent statistical significance between DP and SP subpopulations in the same mouse strain, after evaluating 4 C57BL/6 and 4 NOD thymi.

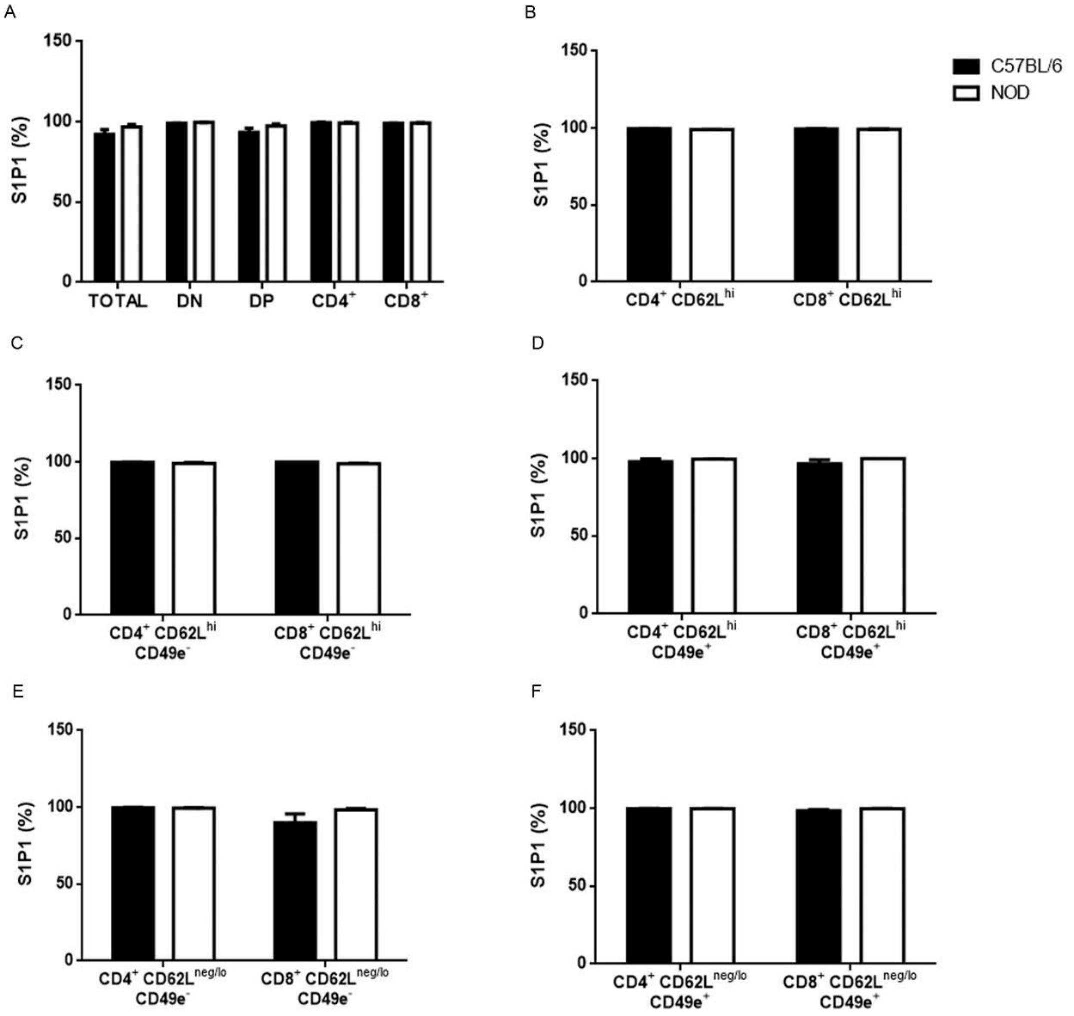


**Figure S4.** Younger NOD mice have no differences in percentage of cells expressing S1P1. The bars show the S1P1 expression on thymocyte subpopulations defined by CD4 and CD8 in C57BL/6 (black bars) and NOD (white bars) mice, analyzed by flow cytometry. (A), S1P1 in total thymocytes, as well as $CD4^{-}CD8^{-}$ double$^{-}$negative, $CD4^{+}CD8^{+}$ double-positive, $CD4^{+}$ and $CD8^{+}$ SP subpopulations, (B), S1P1 in mature $CD4^{+}CD62L^{hi}$ and $CD8^{+}CD62L^{hi}$ SP thymocytes, (C), S1P1 in mature $CD4^{+}CD62L^{hi}CD49e^{-}$ and $CD8^{+}$CD62LhiCD49e$^{-}$ cells, (D), S1P1 in mature $CD4^{+}CD62L^{hi}CD49e^{+}$ and $CD8^{+}CD62L^{hi}CD49e^{+}$ subpopulations, (E), S1P1 in $CD4^{+}CD62L^{neg/lo}CD49e^{-}$ and $CD8^{+}CD62L^{neg/lo}CD49e^{-}$ cells and (F), S1P1 in $CD4^{+}CD62L^{neg/lo}CD49e^{+}$ and $CD8^{+}CD62L^{neg/lo}CD49e^{+}$ subsets. Total = total thymocytes; DN = $CD4^{-}CD8^{-}$ double$^{-}$negative; DP = $CD4^{+}CD8^{+}$ double-positive; $CD4^{+}$. = $CD4^{+}CD8^{-}$ single-positive; $CD8^{+}$ = $CD4^{-}CD8^{+}$ single-positive; % = relative cell numbers. Results are expressed as mean ± SEM and were analyzed by 2way ANOVA followed by Tukey post-test. $n$ = 4 C57BL/6; $n$ = 4 NOD.

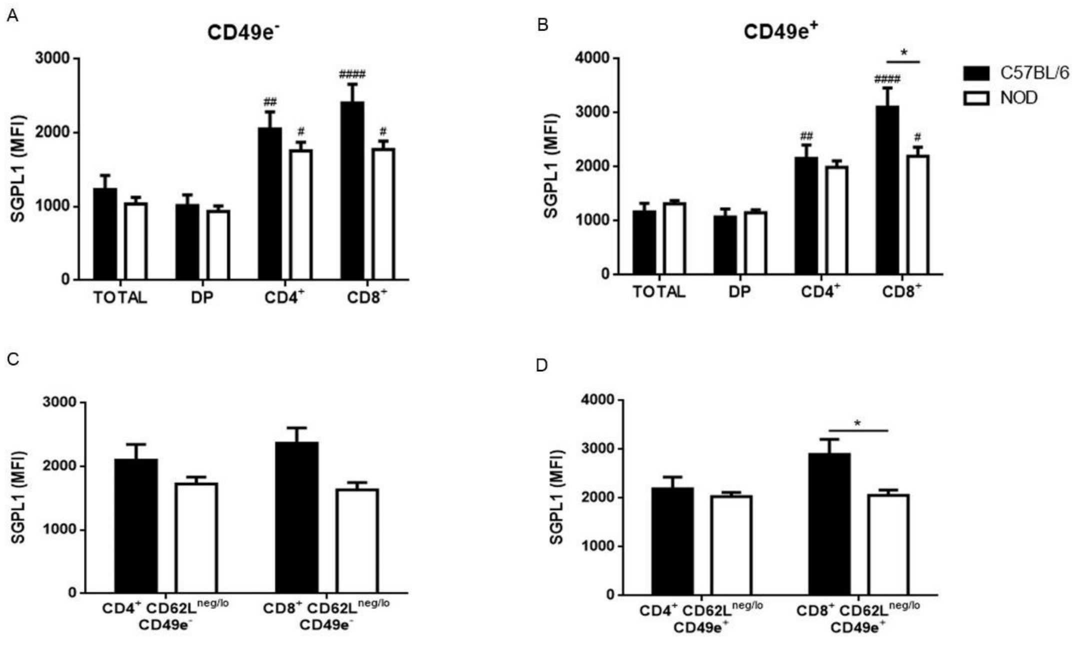


**Figure S5.** NOD CD49e$^+$ and CD49e$^-$ SP thymocytes present lesser SGPL1 expression. The bars show the SGPL1 expression on thymocyte subpopulations defined by CD4 and CD8 in C57BL/6 (black bars) and NOD (white bars) mice, analyzed by flow cytometry. (A), CD49e$^+$ total thymocytes and DP, CD4$^+$ and CD8$^+$ SP subpopulations and (B), CD49e$^-$ total thymocytes and DP, CD4$^+$ and CD8$^+$ SP subpopulations, (C), CD4$^+$ and CD8$^+$ SP CD62L$^{neg/lo}$CD49e$^-$ cells and (D), SP CD4$^+$ and CD8$^+$ CD62L$^{neg/lo}$CD49e$^+$ cells. Total = total thymocytes; DN = CD4$^-$CD8$^-$ double$^-$negative; DP = CD4$^+$CD8$^+$ double-positive; CD4$^+$ = CD4$^+$CD8$^-$ single-positive; CD8$^+$ = CD4$^-$CD8$^+$ single-positive; MFI = median fluorescence intensity. Results are expressed as mean ± SEM and were analyzed by 2way ANOVA followed by Tukey post-test. Differences were considered statistically significant when * or # $p < 0.05$; ## $p < 0.01$; #### $p < 0.0001$. Asterisks represent statistical significance between C57BL/6 and NOD subpopulations; hash marks represent statistical significance between DP and SP subpopulations in the same mouse strain. $n = 8$ C57BL/6; $n = 8$ NOD.

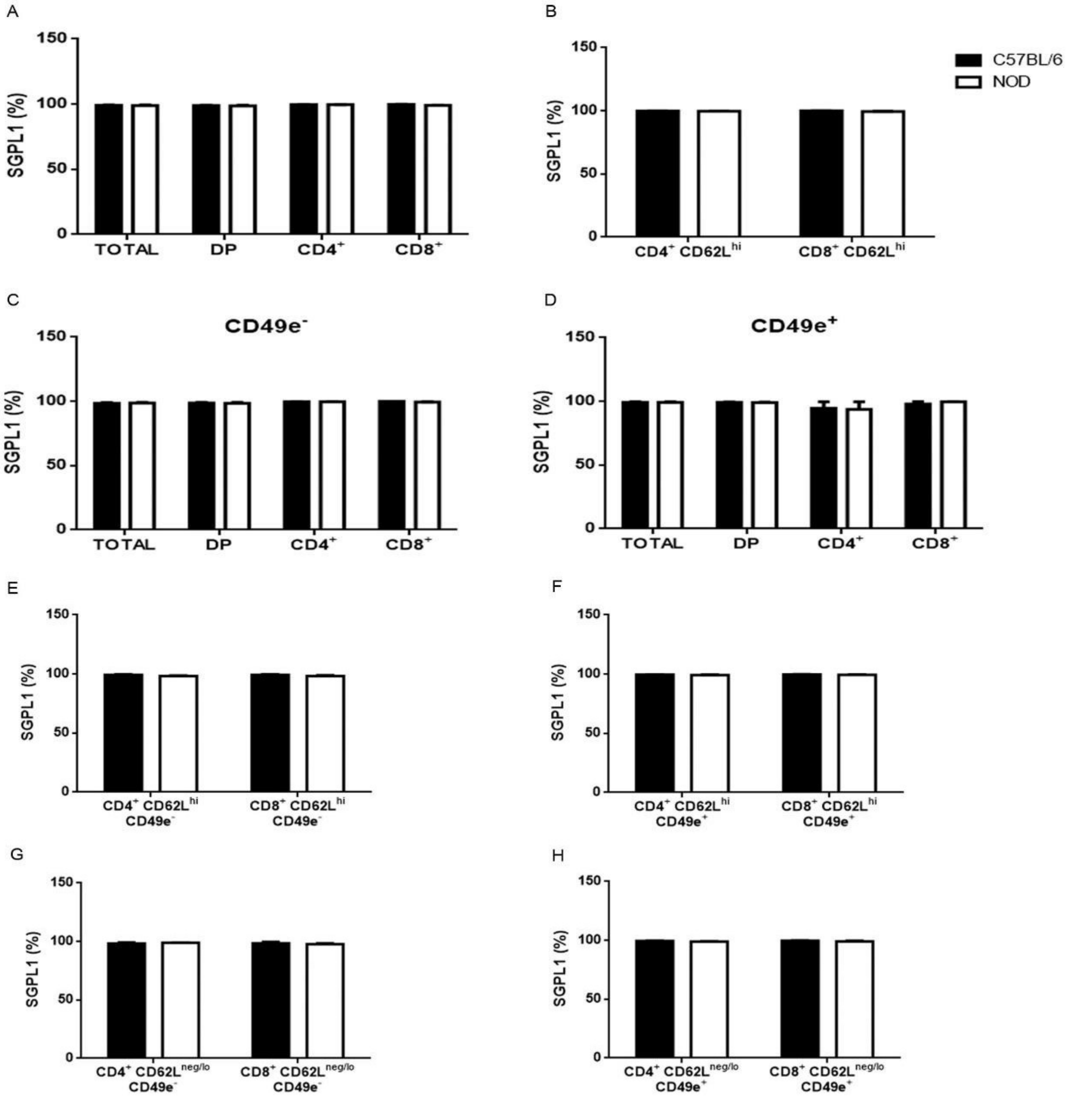


**Figure S6.** NOD mice have similar frequency of cell expressing SGPL1. The bars show the SGPL1 expression on thymocyte subpopulations defined by CD4 and CD8 in C57BL/6 (black bars) and NOD (white bars) mice, analyzed by flow cytometry. (A), SGPL1 in total thymocytes, as well as $CD4^{+}CD8^{+}$ double-positive, $CD4^{+}$ and $CD8^{+}$ SP subpopulations, (B), SGPL1 in mature $CD4^{+}CD62L^{hi}$ and $CD8^{+}CD62L^{hi}$ SP thymocyte subpopulations, (C), SGPL1 in $CD49e^{-}$ total thymocytes and DP, $CD4^{+}$ and $CD8^{+}$ SP subpopulations, (D), SGPL1 in $CD49e^{+}$ total thymocytes and DP, $CD4^{+}$ and $CD8^{+}$ SP subpopulations, (E), SGPL1 in $CD4^{+}$ and $CD8^{+}$ SP $CD62L^{hi}CD49e^{-}$ cells, (F), SGPL1 in SP $CD4^{+}$ and $CD8^{+}$ $CD62L^{hi}CD49e^{+}$ cells, (G), SGPL1 in $CD4^{+}$ and $CD8^{+}$ SP $CD62L^{neg/lo}CD49e^{-}$ cells and (H), SGPL1 in SP $CD4^{+}$ and $CD8^{+}$ $CD62L^{neg/lo}CD49e^{+}$ cells. Total = total thymocytes; DN = $CD4^{-}CD8^{-}$ double-negative; DP = $CD4^{+}CD8^{+}$ double-positive; $CD4^{+}$ = $CD4^{+}CD8^{-}$ single-positive; $CD8^{+}$ = $CD4^{-}CD8^{+}$ single-positive; % = relative cell numbers. Results are expressed as mean ± SEM and were analyzed by 2way ANOVA followed by Tukey post-test. $n$ = 8 C57BL/6; $n$ = 8 NOD.

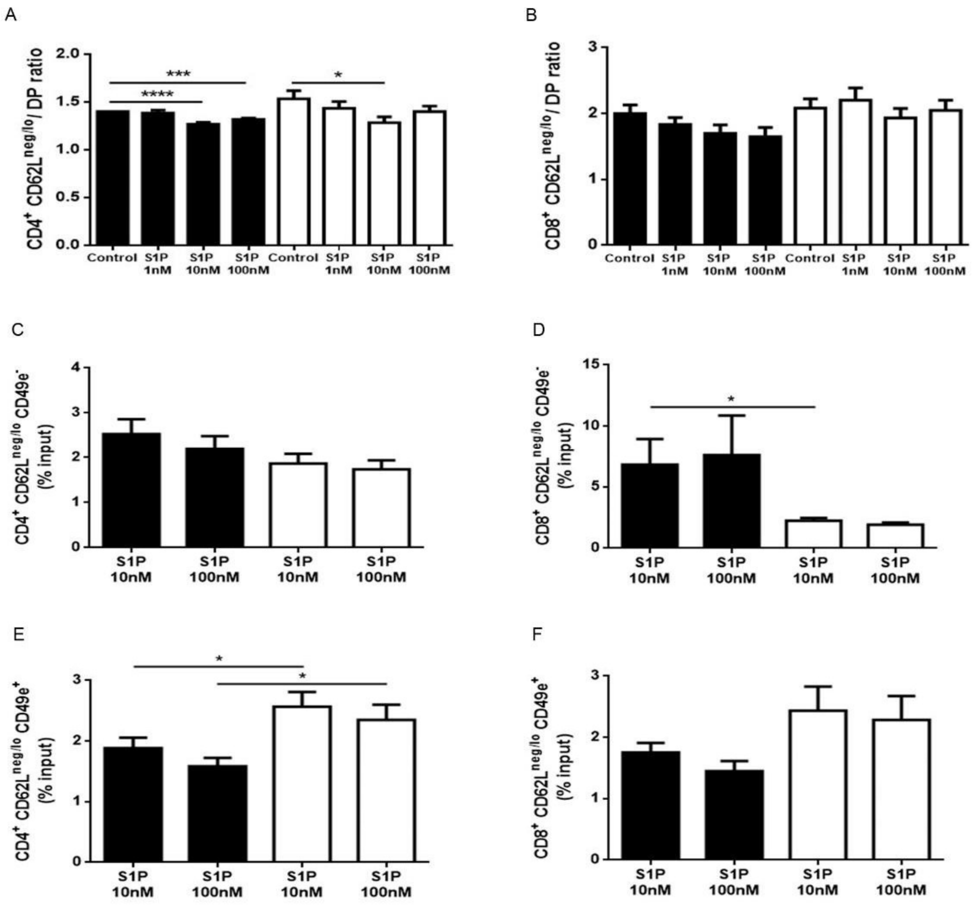


**Figure S7.** CD62L$^{neg/lo}$ thymocyte subpopulations have no chemotactic response under S1P stimulation. The panels show (A), SP CD4$^+$CD62L$^{neg/lo}$, (B), SP CD8$^+$CD62L$^{neg/lo}$, (C), CD4$^+$CD62L$^{neg/lo}$CD49e$^-$, (D), CD8$^+$CD62L$^{neg/lo}$CD49e$^-$, (E), CD4$^+$CD62L$^{neg/lo}$CD49e$^+$ and (F), CD8$^+$CD62L$^{neg/lo}$CD49e$^+$ cells migration towards 1 nM, 10 nM or 100 nM of S1P, in C57BL/6 (black bars) and NOD (white bars) mice. CD4$^+$ = CD4$^+$CD8$^-$ single-positive; CD8$^+$ = CD4$^-$CD8$^+$ single-positive; MFI = median fluorescence intensity. Results are shown as the ratio of the percentages of input (single-positive to double-positive thymocytes) or percentages of input, and are expressed as mean ± SEM, being analyzed by 2way ANOVA followed by Tukey post-test or Student's t test. Differences were considered statistically significant when * $p < 0.05$; *** $p < 0.001$; **** $p < 0.0001$, after evaluating 6 C57BL/6 versus 6 NOD animals.

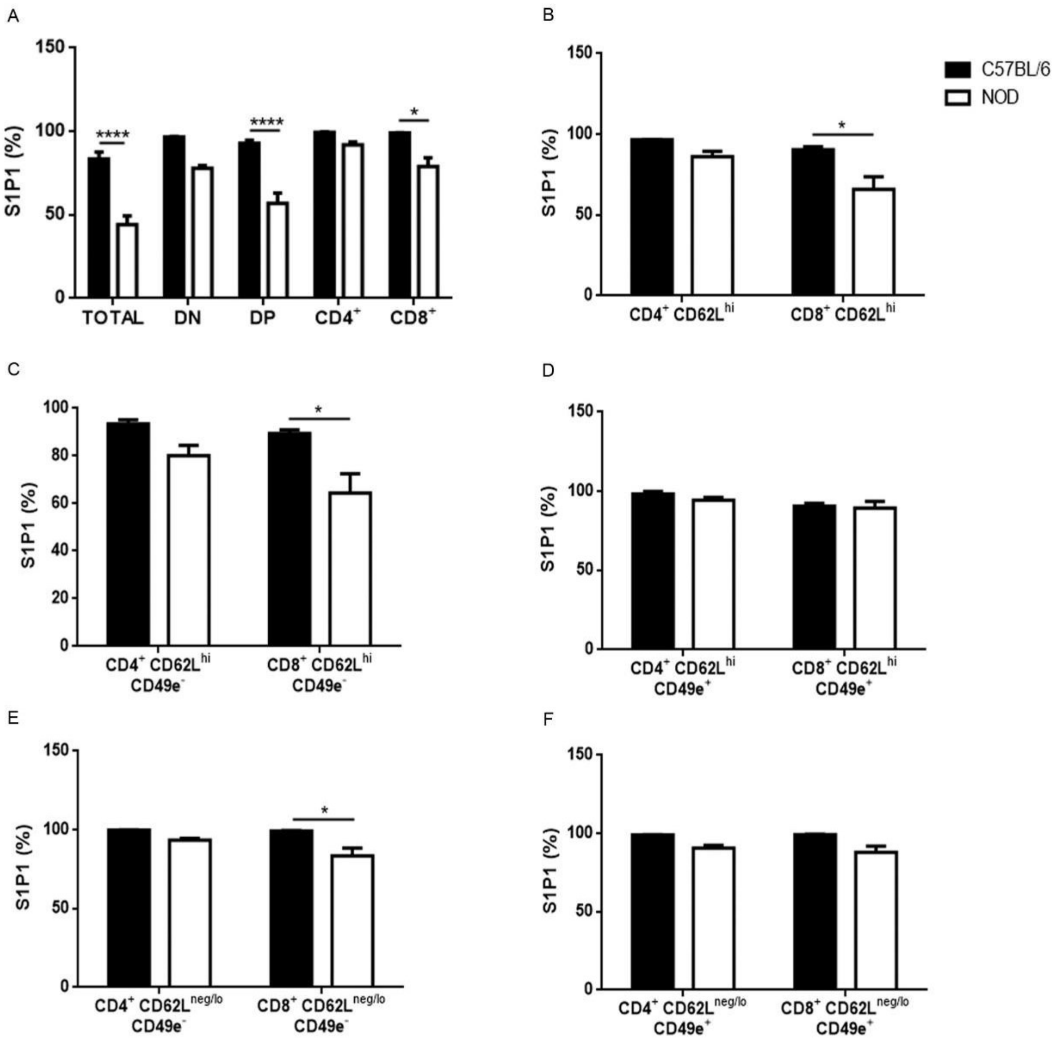


**Figure S8.** NOD mice have decreased percentage of $CD8^+CD49e^-$ cells expressing S1P1. The graphs show the S1P1 expression on thymocyte subpopulations defined by CD4 and CD8 in C57BL/6 (black bars) and NOD (white bars) mice, analyzed by flow cytometry. (A), S1P1 in total thymocytes, as well as $CD4^-CD8^-$ double⁻negative, $CD4^+CD8^+$ double-positive, $CD4^+$ and $CD8^+$ SP subpopulations, (B), S1P1 in mature $CD4^+CD62L^{hi}$ and $CD8^+CD62L^{hi}$ SP thymocyte subpopulations, (C), S1P1 in mature $CD4^+CD62L^{hi}CD49e^-$ and $CD8^+$CD62LhiCD49e⁻ cells, (D), S1P1 in mature $CD4^+CD62L^{hi}CD49e^+$ and $CD8^+CD62L^{hi}CD49e^+$ subpopulations, (E), S1P1 in $CD4^+CD62L^{neg/lo}CD49e^-$ and $CD8^+CD62L^{neg/lo}CD49e^-$ cells and (F), S1P1 in $CD4^+CD62L^{neg/lo}CD49e^+$ and $CD8^+CD62L^{neg/lo}CD49e^+$ subsets. Total = total thymocytes; DN = $CD4^-CD8^-$ $double^-$ negative; DP = $CD4^+CD8^+$ double-positive; $CD4^+$ = $CD4^+CD8^-$ single-positive; $CD8^+$ = $CD4^-CD8^+$ single-positive; % = relative cell numbers. Results are expressed as mean ± SEM and were analyzed by 2way ANOVA followed by Tukey post-test. Differences were considered statistically significant when * $p < 0.05$; **** $p < 0.0001$. $n = 3$ C57BL/6; $n = 4$ NOD.

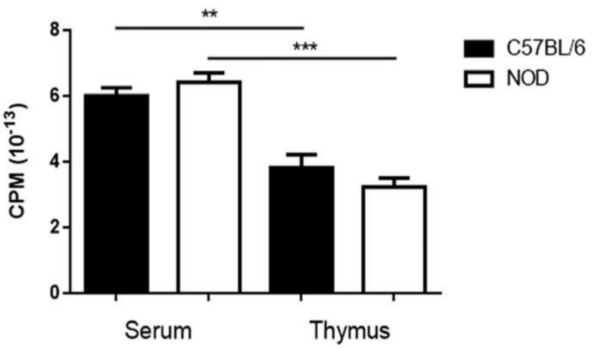


**Figure S9.** NOD mice have normal S1P gradient between thymus and serum. S1P was measured in thymi and sera of C57BL/6 (black bars) and NOD (white bars) by thin-layer chromatography (TLC). Four C57BL/6 and 4 NOD mice were used for these experiments. Results are expressed as mean ± SEM and were analyzed by 2 way ANOVA followed by Tukey post-test. Differences were considered statistically significant when ** $p < 0.01$; *** $p<0.001$. CPM = counts per minute.

**Table S1.** Primers and standard curve parameters for gene amplification in thymi from C57BL/6 and NOD mice.

| Mouse gene target | Primer sequences (5'-3') | Slope | Intercept | Coefficient of linearity ($r^2$) | Amplification efficiency (%) |
|---|---|---|---|---|---|
| | | | | | |
| **S1P1** | FW - GTGTAGACCCAGAGTCCTGCG | -3,06 | 15,9 | 0,99 | 112,4 |
| | RV - AGCTTTTCCTTGGCTGGAGAG | | | | |
| **HPRT** | FW - TCCCAGCGTCGTGATTAGCGATG | -2,89 | 13,96 | 0,98 | 121,8 |
| | RV - GGCCACAATGTGATGGCCTCCC | | | | |
| **GAPDH** | FW - CCACTCACGGCAAATTCAACGGC | -3,16 | 9,62 | 0,98 | 107,2 |
| | RV - CCACCCTTCAAGTGGGCCCCG | | | | |
